# Estimated Incidence of Seasonal Influenza in China From 2010 to 2020 Using a Multiplier Model

**DOI:** 10.1001/jamanetworkopen.2022.7423

**Published:** 2022-04-14

**Authors:** Qiang Wang, Liuqing Yang, Chang Liu, Hui Jin, Leesa Lin

**Affiliations:** 1 Department of Epidemiology and Health Statistics, School of Public Health, Southeast University, Nanjing, China; 2 Key Laboratory of Environmental Medicine Engineering, Ministry of Education, School of Public Health, Southeast University, Nanjing, China; 3 Department of Infectious Disease Epidemiology, London School of Hygiene & Tropical Medicine, London, United Kingdom; 4 Laboratory of Data Discovery for Health, Hong Kong Science Park, Hong Kong, China

## Abstract

This quality improvement study estimates the total incidence of seasonal influenza and associated illnesses in China from 2010 to 2020.

## Introduction

Understanding influenza burden is crucial for providing a reference for public health decision-making. According to Reed et al,^1^ the reported number of influenza cases is much less than the true number of cases. We aimed to estimate the true number of individuals with influenza virus infections, symptomatic influenza illnesses, and influenza-associated medically attended illnesses in China between 2010 and 2020, and then estimate the incidence rates for these outcomes.

## Methods

**Figure 1.**
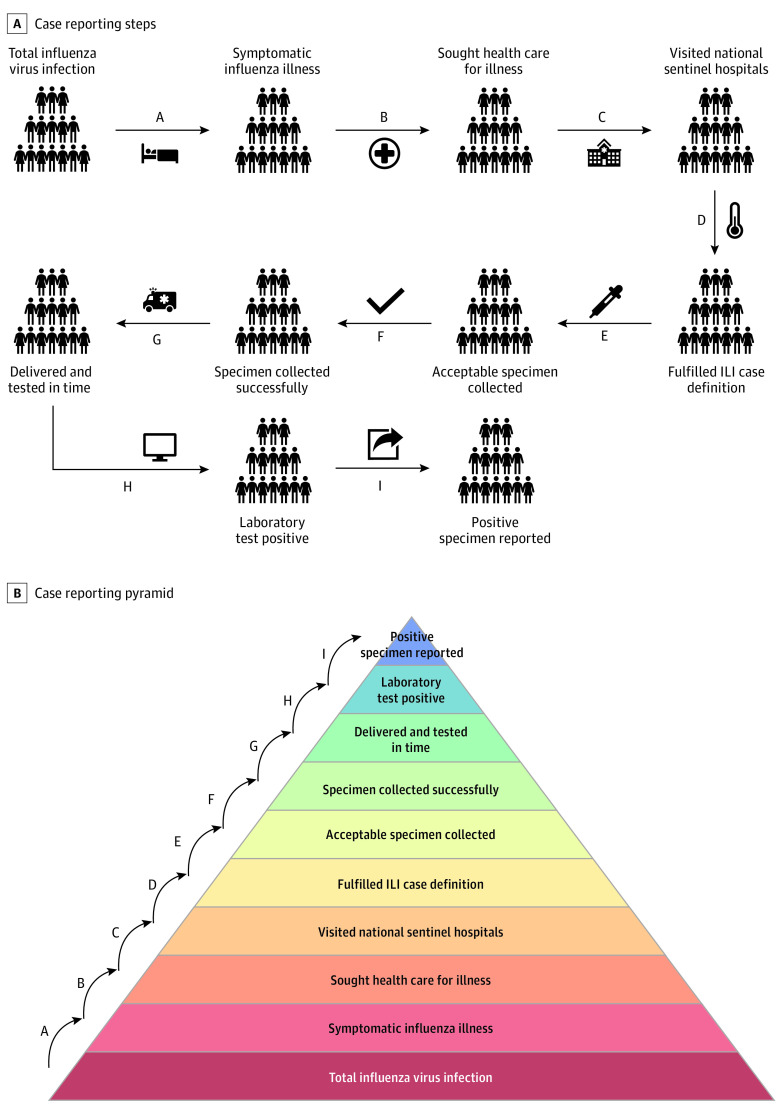
Schematic of the Steps ILI indicates influenza-like illness.

Using data from the Chinese surveillance program,^2,3^ our quality improvement study used the multiplier model method described by Reed et al^1^ and made further improvements based on the revised model offered by Wu et al^4^ to construct an estimate of total influenza cases given the small proportion of the total number of influenza virus infections that are identified and reported after a series of medical surveillance processes (Figure 1; eMethods, eFigures 1 through 3 in the Supplement). Parameters were derived from meta-analyses, previous studies, professional consultations, influenza weekly reports, and statistical yearbooks (eTable in the Supplement). This study was exempt from ethics review by the Southeast University ethics review board because it did not use human participants, and informed consent requirements were waived because data were deidentified. This study followed Standards for Quality Improvement Reporting Excellence (SQUIRE) reporting guideline for quality improvement studies.

The number of infections was estimated by age for children (ages 0 to 14 years), younger adults (15 to 59 years), and older adults (60 years or older). The parameters were assigned a uniform probability distribution, and 10 000 Monte Carlo simulations were performed of the calculations.^1^ The median values and the 95% uncertainty intervals (UI; percentiles 2.5 to 97.5) of the number of infections, symptomatic illnesses (SI), and symptomatic individuals seeking health care (ie, medically attended illnesses [MAI]) in each influenza season were calculated and reported. The incidence of infections, SI, and MAI per 1000 persons were estimated by dividing the number of these outcomes by the size of the population. All analyses were performed using Stata version 14.0 (StataCorp) and Microsoft Excel 2016 (Microsoft Corporation).

## Results

**Figure 2.**
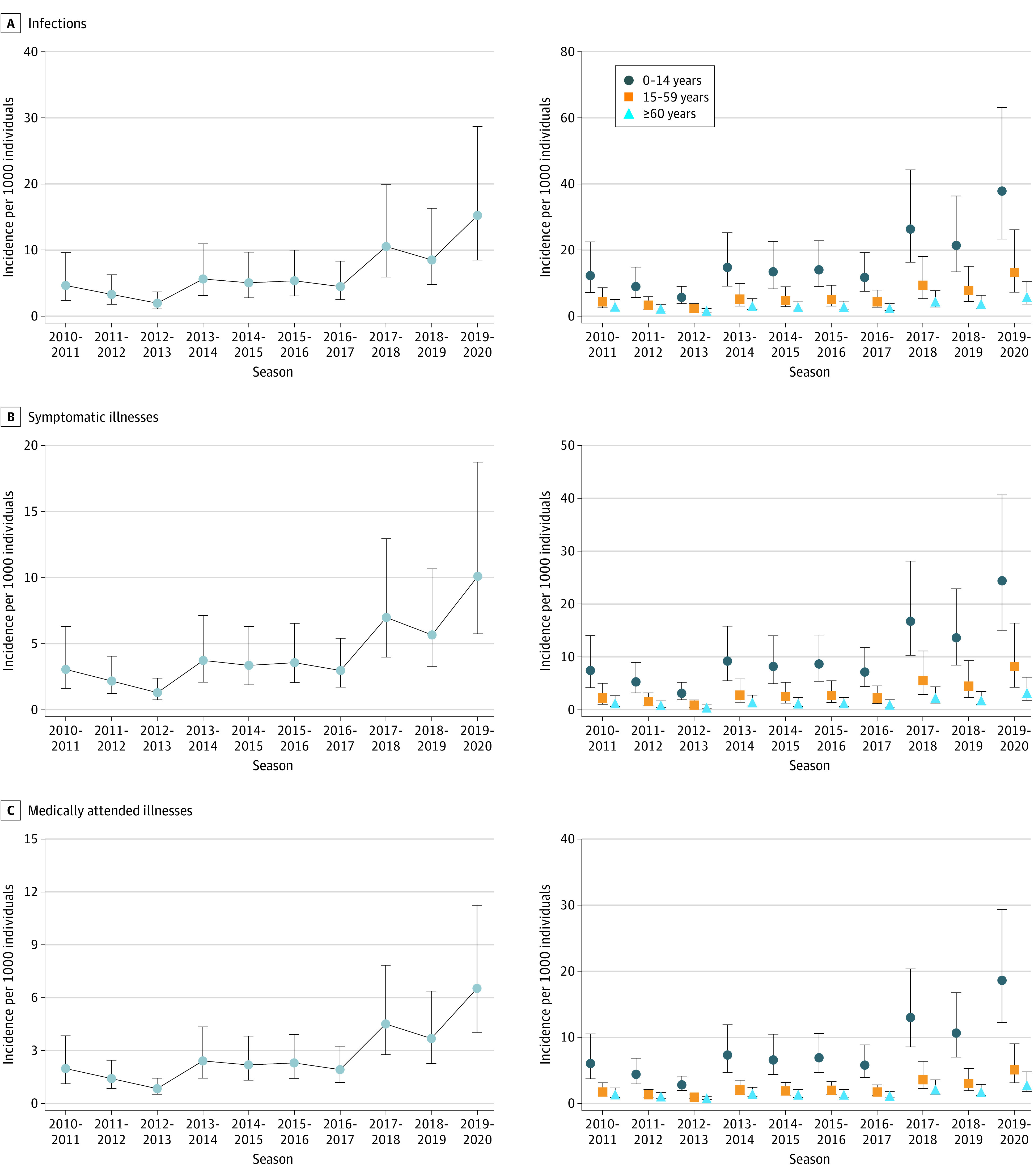
Estimated Incidence of Infections, Symptomatic Illnesses, and Medically Attended Illnesses by Age Group and Season Whiskers indicate 95% UI.

Between 2010 and 2020, the cumulative total number of influenza virus infections, SI, and MAI were 89 452 622 (95% UI, 49 896 796-170 503 669), 59 215 754 (95% UI, 33 655 540-111 064 340) and 38 414 517 (95% UI, 23 419 932-67 030 271), respectively. The mean incidence rate of infections, SI, and MAI per 1000 person-seasons was 6.48 (95% UI, 3.61-12.36), 4.29 (95% UI, 2.44-8.05), and 2.78 (95% UI, 1.70-4.86), respectively (Figure 2).

Between 2010 and 2020, the annual mean number of infections was 3 646 403 (95% UI, 2 200 888-6 259 352) for individuals aged 0 to 14 years, 4 762 049 (95% UI, 2 490 075-9 757 261) for those aged 15 to 59 years, and 536 811 (95% UI, 298 717-1 033 753) for those 60 years or older. In the children cohort, the annual mean incidence rate of infections, SI, and MAI per 1000 person-seasons was 15.86 (95% UI, 9.57-27.23), 10.50 (95% UI, 6.45-17.68), and 7.81 (95% UI, 5.02-12.57), respectively. In the 60 years or older cohort, the annual mean incidence rate of infections, SI, and MAI per 1000 person-seasons was 2.37 (95% UI, 1.32-4.57), 1.57 (95% UI, 0.89-2.98), and 1.12 (95% UI, 0.65-2.08), respectively.

## Discussion

Our study estimated that there were approximately 50 to 170 million individuals with influenza virus infections in China between 2010 and 2020. We found that 1 positive test sample reported was likely to represent 282 individuals with influenza virus infections, 187 with symptomatic illnesses, and 121 with influenza-associated illnesses who sought health care in China. The estimated incidence of symptomatic infections in China was much lower than that estimated in the US (approximately 100 per 1000 persons per year).^5^ According to our estimates, the incidence rate of influenza per 1000 persons was highest among children, being 3.1 times and 6.6 times that of younger adults and older adults, respectively.

This study did have limitations. Although we estimated all model inputs as accurately as possible, considerable uncertainty in these parameters and therefore in the overall results remains. Additionally, some parameters in February and March 2020 might have differed from previous seasons due to the COVID-19 epidemic and China’s lockdown policy.

This study provided a tool to estimate seasonal influenza burden and appealed to enhance influenza surveillance ability in China. The reported number of influenza cases was considerably less than the true number of cases.

## Notes

Supplement.
eMethods.
**eTable.** Estimated Parameters in the Model **eFigure 1.** Flowchart of Study Selection for Proportion B **eFigure 2.** Flowchart of Study Selection for Proportion C **eFigure 3.** Flowchart of Study Selection for Proportion E
