# Supplementary material for: Estimated Incidence of Seasonal Influenza in China From 2010 to 2020 Using a Multiplier Model
Source: JAMA Netw Open. 2022 Apr 14;5(4):e227423. doi: 10.1001/jamanetworkopen.2022.7423 (PMC9011120; doi:10.1001/jamanetworkopen.2022.7423)
Supplement: Supplement. — eMethods. eTable. Estimated Parameters in the Model eFigure 1. Flowchart of Study Selection for Proportion B eFigure 2. Flowchart of Study Selection for Proportion C eFigure 3. Flowchart of Study Selection for Proportion E [file jamanetwopen-e227423-s001.pdf]

Supplemental Online Content

Wang Q, Yang L, Liu C, Jin H, Lin L. Estimated incidence of seasonal influenza in China from 2010 to 2020 using a multiplier model. *JAMA Netw Open.* 2022;5(4):e227423. doi:10.1001/jamanetworkopen.2022.7423

eMethods.

eTable. Estimated Parameters in the Model

eFigure 1. Flowchart of Study Selection for Proportion B

eFigure 2. Flowchart of Study Selection for Proportion C

eFigure 3. Flowchart of Study Selection for Proportion E

This supplemental material has been provided by the authors to give readers additional information about their work.

© 2022 Wang Q et al. *JAMA Network Open.*

eMethods

Surveillance program and model structure

The Chinese health authorities developed an influenza surveillance system in 2000 by setting-up a national network of sentinel surveillance hospitals to monitor and report cases of ILI. As of 2010, there were 556 national sentinel surveillance hospitals in China.[1] The surveillance departments in sentinel surveillance hospitals include internal medicine clinics (or pediatric medicine clinics), internal medicine emergency departments (or pediatric medicine emergency departments), and fever clinics. The staffs in these departments diagnose cases of ILI according to a set definition (fever, i.e., a body temperature ≥38°C, accompanied by cough or sore throat), and record the number of ILI cases and the total number of consultations in the department each day. These data are uploaded to the China Influenza Surveillance Information System (CISIS) every Monday.[2] Nasopharyngeal swabs are collected from one or two patients with ILI each day and delivered to the influenza surveillance network of laboratories for testing. Approximately 20 samples (range: 10–40 samples) are collected and delivered for testing per week. This meant that only a small proportion of ILI cases got laboratory tests. Hemagglutination inhibition and/or real-time reverse-transcription polymerase chain reaction (PCR) assays are used to identify influenza viruses in the samples. The test results are uploaded to CISIS within 48 hours of testing.

According to the Chinese surveillance program, we used the multiplier model method described by Reed et al [3]and Wu et al.[4] Only a small proportion of the total number of influenza virus infections are identified and reported after a series of processes. The true total number of infections was estimated by dividing the number of positive specimens by nine proportions, described below.

Parameters

Nine proportions are needed in the model, including the proportion of symptomatic cases among the total number of influenza virus infections (A); proportion of individuals with symptomatic infections seeking health care (B); proportion of symptomatic illnesses seeking health care seen at national sentinel hospitals (C); proportion of individuals with influenza who fulfilled the definition of ILI (D); proportion of patients with ILI that accept specimen collection (E); proportion of specimens collected successfully (F); proportion of specimens delivered and tested in a timely manner (G); test sensitivity (H); and proportion of positive specimens reported in a timely manner (I) (eTable 1).

A and H were based on previous systematic reviews.[5,6] B, C, and E were obtained through meta-analyses that pooled data after performing a literature search of the Chinese and English literature. For B, ten of 60 studies reviewed in full text provided data about the likelihood of individuals with ILI seeking health care, and the extracted data were pooled according to age group to obtain age-specific estimates. The pooled rate among individuals aged 0–14 years, 15–59 years, and ≥60 years was estimated to be 76.03%, 58.14%, and 72.00%, respectively. In the process of finding and extracting C, 2,450 articles were identified, and the full texts of 50 articles were reviewed. Finally, only one study in Suzhou city was used to obtain estimates of the proportion of individuals with ILI that sought health care at national sentinel hospitals who were aged between 0–14 years, and the rate was 28.32% (192/678).[7] Given the high economic level of development in Suzhou city, we used 20%-30% as a proxy range of C among individuals aged 0-14 years. Most national sentinel hospitals are municipal hospitals.[1,2] A national study in the 2017–2018 season reported that the proportions of symptomatic individuals that sought health care at municipal hospitals were 27.01% and 26.62% among individuals aged 15–59 years and ≥60 years, respectively.[8] We multiplied these values by 0.4 to 0.9, as the range of C among the 15–59 year and ≥60 year age groups. For E, a total of 2,330 articles were searched, of

which 492 repeated articles and 1,394 unrelated articles were excluded, and 69 articles were finally included after reviewing 444 articles. The proportion of individuals with ILI that accepted specimen collecting was pooled by season. The pooled rates ranged from 10.34% to 23.84% across the ten influenza seasons between 2010 and 2020. Search strategies and flowcharts of the meta-analyses are shown below (eFigure 1-3). D and F were derived from a previous multiplier model study.[9] G and I were obtained from the consultations of healthcare workers in the Center for Disease Control and Prevention and influenza weekly reports. The number of positive specimens was derived from the influenza weekly report published by the Chinese National Influenza Center (http://www.chinaivdc.cn/cnic/). Information about the population size and age-specific population size in China by year was obtained from the China Statistical Yearbook (http://www.stats.gov.cn/tjsj/ndsj/).

The influenza weekly report did not provide the percentage of positive specimens according to age, so we used the parameter from an epidemiologic study conducted in Jiangsu province, China, between 2010 and 2014.[10] The proportions were 56.38%, 38.31%, and 5.31% in the 0–14 year, 15–59 year, and ≥60 year age groups, respectively.

Data Analysis

The number of infections was estimated for the following age groups: children (0–14 years), younger adults (15–59 years), and older adults (≥60 years). The number of cases of influenza was estimated during each influenza season between 2010 and 2020. The influenza season was defined as starting in the 14th week of each year and ending in the 13th week of the following year.[1] Assumptions in the model included:

a) Except for E, the parameters in the model were assumed to be the same in different influenza seasons because the available data were not sufficient to estimate the parameters according to the season.
b) Except for B and C, different age groups were assumed to share the same parameters.
c) Except for H, the parameters in the model were assumed to be the same for influenza A virus and influenza B virus.

The parameters were assigned a uniform probability distribution, and 10,000 Monte Carlo simulations were performed of the calculations.[3] Values in the range between the minimum and maximum values of each parameter were randomly sampled for 10,000 iterations. The median values and the 95% uncertainty range (UR) (centiles 2.5 to 97.5) of the number of influenza virus infections, number of symptomatic influenza illnesses, and influenza-associated individuals with symptoms seeking health care (medically attended) in each influenza season were calculated and reported. The incidence of influenza virus infection, symptomatic illnesses (SI), and medically attended illnesses (MAI) per 1,000 persons were estimated by dividing the number of these outcomes by the size of the population. Additionally, the parameters were assigned a normal probability distribution, and the median values and 95% UR of number of infections, SI, and MAI were reported after 10,000 iterations. The estimates were similar when estimated using a uniform probability distribution and a normal distribution. Moreover, to assess model accuracy and reliability, we calculated the results using the adapted model and compared them with the estimates Wu et al [4] calculated in a previous study conducted in Beijing, China. Wu et al [4] estimated the number of influenza virus infection in Beijing in the 2015-2016 season to be 1,190,200 (95% confidence interval: 830,400 to 1,549,900). Using the adapted method, the infection was estimated to be 817,287 (95% UR: 451,173 to 1,587,945) using a uniform probability distribution and 861,782 (95% UR: 514,507 to 1,571,449) using a normal distribution. The meta-analyses were performed using Stata version 14.0 (StataCorp, College Station,

TX, USA) and Monte Carlo probability simulations were performed using Microsoft Excel 2016 (Microsoft, Redmond, WA, USA). This study followed the SQUIRE reporting guidelines. We provided more detailed results in http://dx.doi.org/10.13140/RG.2.2.13121.22888.

Reference

1. Shu Y, Song Y, Wang D, et al. A ten-year China-US laboratory collaboration: improving response to influenza threats in China and the world, 2004-2014. *BMC Public Health*. 2019;19(Suppl 3):520. Published 2019 May 10. doi:10.1186/s12889-019-6776-3
2. Chinese National Influenza Center. National Influenza Surveillance Technical Guide (2017 Edition). Accessed December 1,2021. http://ivdc.chinacdc.cn/cnic/zyzx/jcfa/201709/t20170930_153976.htm.
3. Reed C, Angulo FJ, Swerdlow DL, et al. Estimates of the prevalence of pandemic (H1N1) 2009, United States, April-July 2009. *Emerg Infect Dis*. 2009;15(12):2004-2007. doi:10.3201/eid1512.091413
4. Wu S, VAN Asten L, Wang L, et al. Estimated incidence and number of outpatient visits for seasonal influenza in 2015-2016 in Beijing, China. *Epidemiol Infect*. 2017;145(16):3334-3344. doi:10.1017/S0950268817002369
5. Carrat F, Vergu E, Ferguson NM, et al. Time lines of infection and disease in human influenza: a review of volunteer challenge studies. *Am J Epidemiol*. 2008;167(7):775-785. doi:10.1093/aje/kwm375
6. Huang HS, Tsai CL, Chang J, Hsu TC, Lin S, Lee CC. Multiplex PCR system for the rapid diagnosis of respiratory virus infection: systematic review and meta-analysis. *Clin Microbiol Infect*. 2018;24(10):1055-1063. doi:10.1016/j.cmi.2017.11.018
7. Zhang T, Zhang J, Hua J, et al. Influenza-associated outpatient visits among children less than 5 years of age in eastern China, 2011-2014. *BMC Infect Dis*. 2016;16:267. Published 2016 Jun 10. doi:10.1186/s12879-016-1614-z
8. Ren X, Geoffroy E, Tian K, et al. Knowledge, Attitudes, and Behaviors (KAB) of Influenza Vaccination in China: A Cross-Sectional Study in 2017/2018. *Vaccines (Basel)*. 2019;8(1):7. Published 2019 Dec 26. doi:10.3390/vaccines8010007
9. Wang X, Yang P, Seale H, et al. Estimates of the true number of cases of pandemic (H1N1) 2009, Beijing, China. *Emerg Infect Dis*. 2010;16(11):1786-1788. doi:10.3201/eid1611.100323
10. Zi HR. Influenza Surveillance and Molecular Epidemiology of Influenza A /H1N1 (09pdm) viruses, Jiangsu province,2010-2014. Southeast University (master dissertation).2015.

© 2022 Wang Q et al. *JAMA Network Open.*

eTable 1. Estimated parameters in the model

Parameter	Range	Source
A: Proportion of total of influenza virus infection that are symptomatic	58.3%, 74.55%	[5]
B: Proportion of individuals with symptomatic infection who seek care	0-14 years: 61.09%, 88.28%<br>15-59 years: 42.22%, 73.25%<br>≥60 years: 61.44%, 81.51%	Meta analysis
C: Proportion of symptomatic illness seeking health care seen at national sentinel hospitals	0-14 years: 20.00%, 30.00%<br>15-59 years: 10.81%, 24.31%<br>≥60 years: 10.65%, 23.96%	[7,8]
D: Proportion of individuals with influenza who fulfilled the definition of influenza ill-likeness	26%, 42%	[9]
E: Proportion of individuals with an influenza ill-likeness who accepted specimen collection	2010-2011: 6.77%, 14.55%<br>2011-2012: 9.20%, 14.66%<br>2012-2013: 13.93%, 20.82%<br>2013-2014: 12.14%, 20.87%<br>2014-2015: 13.56%, 22.07%<br>2015-2016: 19.08%, 27.83%<br>2016-2017: 19.24%, 28.78%<br>2017-2018: 15.60%, 24.24%<br>2018-2019: 13.78%, 21.28%<br>2019-2020: 9.08%, 13.86%	Meta analysis
F: Proportion of specimens that were collected successfully	80%, 90%	[9]
G: Proportion of specimens that were delivered and tested timeously	90%, 100%	Professional consultations and influenza weekly report
H: Test sensitivity	influenza A: 90.2%, 96.4%<br>influenza B: 87.7%, 96.3%	[6]
I: Proportion of positive influenza test results reported timeously	90%, 100%	Professional consultations and influenza weekly report

© 2022 Wang Q et al. *JAMA Network Open.*

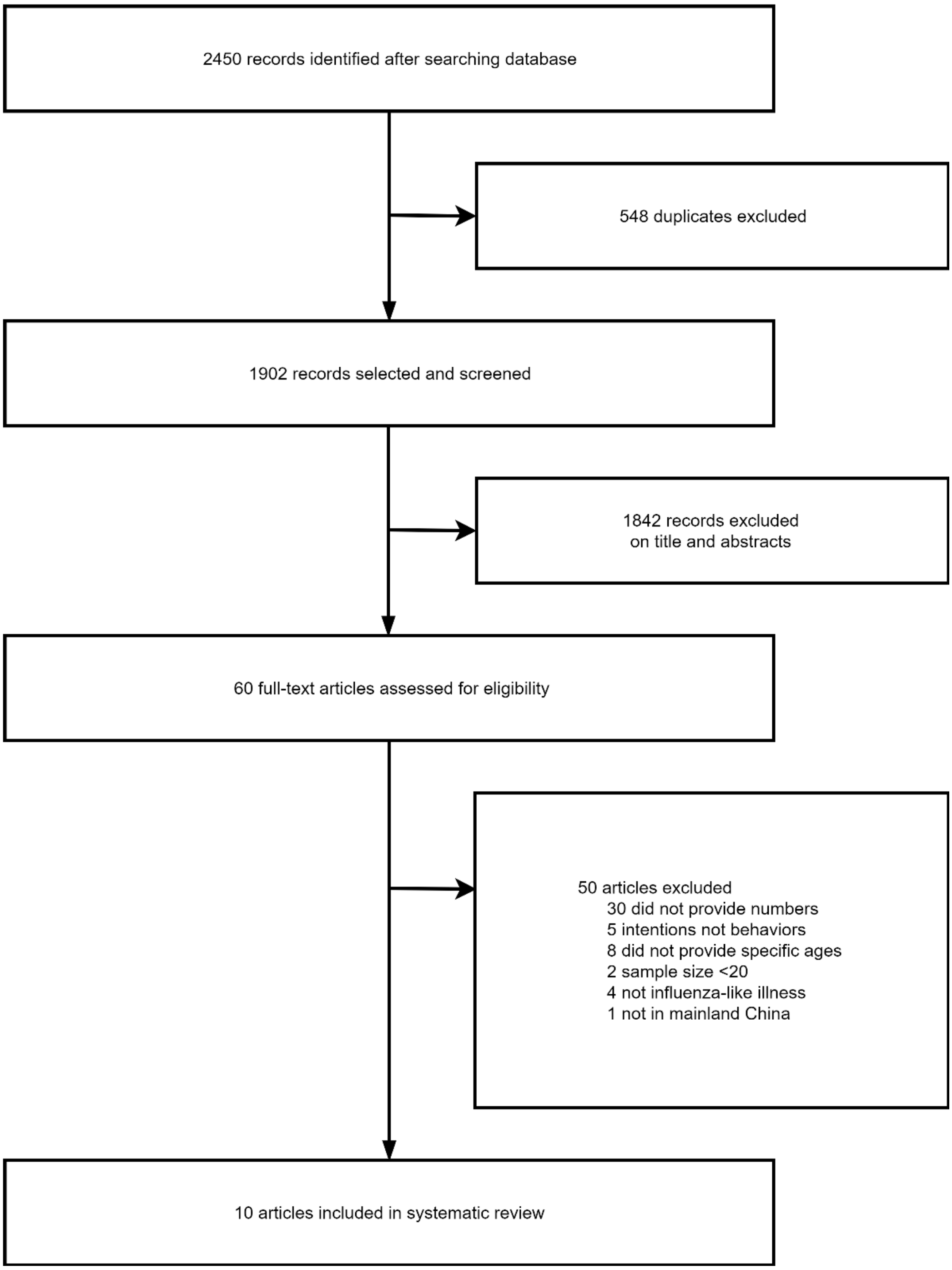

eFigure 1. Flowchart of study selection for proportion B[a]

[a] search strategy: We used the following search terms: “influenza” OR “influenza like illness” OR “influenza-like illness” OR “ILI” AND “seeking behaviour*” OR “seek” AND “China”. A systematic search of the available medical literature was performed on October 07, 2021, using Chinese language databases including China National Knowledge Infrastructure, Chinese Science and Technology Periodical Database, and WanFang Database, and English language databases including PubMed, and Web of Science.

© 2022 Wang Q et al. *JAMA Network Open.*

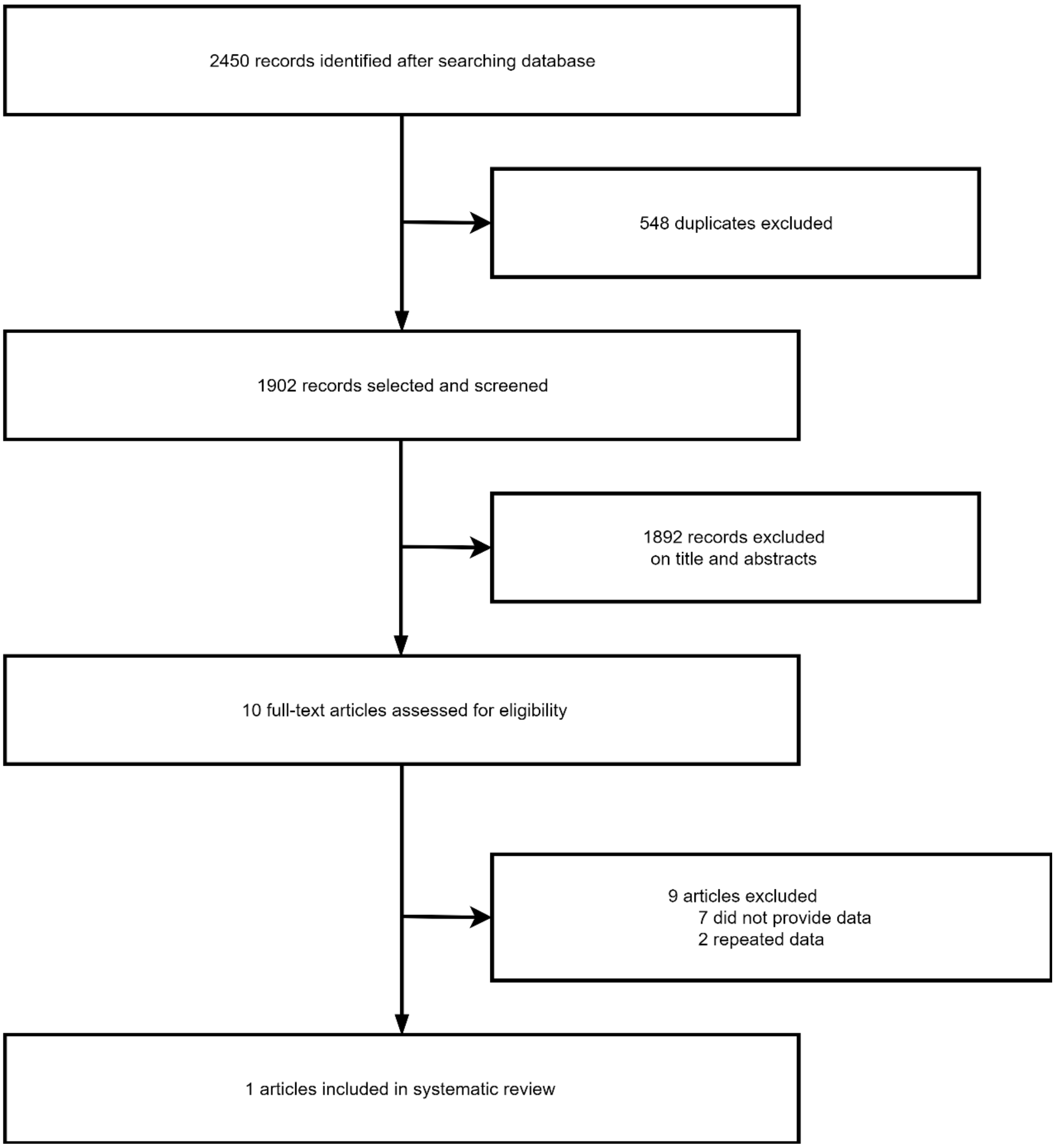

eFigure 2. Flowchart of study selection for proportion C[a]

[a] search strategy: We used the following search terms: “influenza” OR “influenza like illness” OR “influenza-like illness” OR “ILI” AND “seeking behaviour*” OR “seek” AND “China”. The systematic search of the available medical literature was performed on October 07, 2021, using Chinese language databases including China National Knowledge Infrastructure, Chinese Science and Technology Periodical Database, and WanFang Database, and English language databases including PubMed, and Web of Science.

© 2022 Wang Q et al. *JAMA Network Open.*

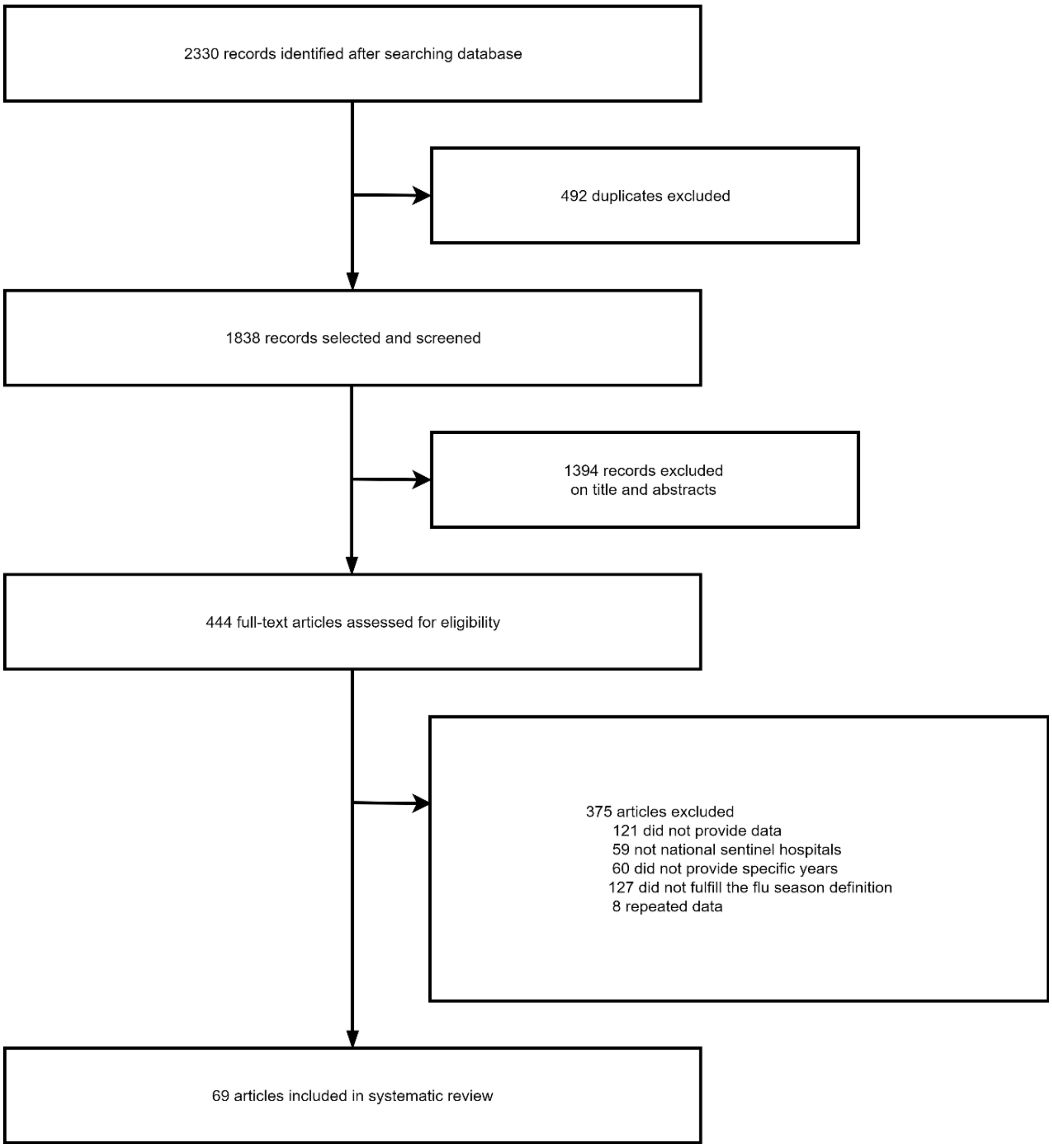

eFigure 3. Flowchart of study selection for proportion E[a]

[a] search strategy We used the following search terms: "influenza like illness" OR "influenza-like illness" OR "ILI" AND "China". A systematic search of the available medical literature was performed on September 19, 2021, using Chinese language databases including China National Knowledge Infrastructure, Chinese Science and Technology Periodical Database, and WanFang Database, and English language databases including PubMed, and Web of Science.

© 2022 Wang Q et al. *JAMA Network Open.*
